# Diagnostic Stability and Predictive Factors of Acute and Transient Psychotic Disorders: A Retrospective Study from Turkey

**DOI:** 10.1192/j.eurpsy.2025.627

**Published:** 2025-08-26

**Authors:** I. Yelli, B. Koparal, S. Özcelep, M. H. Aksu

**Affiliations:** 1 Department of Psychiatry, Gazi University Faculty of Medicine, Ankara, Türkiye

## Abstract

**Introduction:**

The 10th edition of the International Classification of Diseases (ICD-10) categorizes acute and transient psychotic disorders (ATPD) as a composite group that merges traditional acute psychosis definitions, characterized by acute onset (≤15 days) and rapid resolution (1-3 months), often linked to stress (WHO, 1992). The incidence of ATPD ranges from 4-10 per 100,000, with a prevalence between 5.8% and 19% (Marneros *et al.* Cambridge University Press 2004; Queirazza *et al.* BJPsych 2014; 204:299-305), predominantly affecting middle-aged to older individuals and females. Diagnostic stability for ATPD varies, with reported values between 35.9% and 56% (Aadamsoo *et al.* Nord. J. Psychiatry 2011; 65:381-8; Poon *et al.* Nord. J. Psychiatry; 2017; 71:139-44), influenced by factors such as age of onset, gender, comorbid substance use and symptom overlap (Taş *et al.* Noro Psikiyatr Ars 2019; 56:47-51). Studies indicate that the highest diagnostic consistency is linked to schizophrenia diagnoses (Queirazza *et al.* BJPsych 2014; 204:299-305).

**Objectives:**

This study retrospectively investigates the temporal stability of first-episode ATPD over a three-year follow-up period. Furthermore, it aims to explore whether initial clinical presentations and sociodemographic factors may serve as prognostic indicators for diagnostic transitions to schizophrenia or other psychotic disorders.

**Methods:**

Patients diagnosed with ATPD and followed over 3 years were evaluated retrospectively. Socio-demographic and clinical variables potentially associated with diagnostic transitions were investigated. The study was approved by the Gazi University School of Medicine Ethics Committee (2023-889).

**Results:**

106 patients (57 males, 49 females) with a mean age of 29.90 ± 10.33 years were included. The diagnostic stability of ATPD was found to be 17.8%. Additionally, 62.3% of the subjects received a diagnosis of schizophrenia or other psychotic disorders. Significant differences were observed between diagnostic groups with regard to education level (χ² = 9.776, p = 0.008) and hospitalization rates (χ² = 8.083, p = 0.018). Multivariate logistic regression analysis indicated that younger age at onset (OR = 0.951, 95% CI 0.90−0.96; p = 0.032) and lower educational attainment (OR = 0.219, 95% CI 0.08−0.54; p = 0.001) were significantly associated with a diagnostic shift to schizophrenia or other psychotic disorders.

**Conclusions:**

In Turkey, the diagnostic stability of ATPD is notably low, with the most common diagnostic shift being towards schizophrenia-related disorders. To our knowledge, no comprehensive studies have been conducted in Turkey that evaluate patients diagnosed with ATPD. Therefore, our study aims to contribute to the existing literature. Further research involving larger sample sizes is needed to assess the influence of clinician attitudes and stigma on diagnostic changes.

**Disclosure of Interest:**

None Declared

